# Comparison of Multiple Clinical Testing Modalities for Assessment of NPM1-Mutant AML

**DOI:** 10.3389/fonc.2021.701318

**Published:** 2021-08-30

**Authors:** Amanda Lopez, Sanjay Patel, Julia T. Geyer, Joelle Racchumi, Amy Chadburn, Paul Simonson, Madhu M. Ouseph, Giorgio Inghirami, Nuria Mencia-Trinchant, Monica L. Guzman, Alexandra Gomez-Arteaga, Sangmin Lee, Pinkal Desai, Ellen K. Ritchie, Gail J. Roboz, Wayne Tam, Michael J. Kluk

**Affiliations:** ^1^Department of Pathology and Laboratory Medicine, Weill Cornell Medicine, New York, NY, United States; ^2^Clinical and Translational Leukemia Program, Division of Hematology and Oncology, Department of Medicine, Weill Cornell Medicine, New York, NY, United States; ^3^Stem Cell Transplant Program, Division of Hematology and Oncology, Department of Medicine, Weill Cornell Medicine, New York, NY, United States

**Keywords:** NPM1, AML, MRD, RT-PCR, NGS, IHC, Flow Cytometry

## Abstract

**Background:**

*NPM1* mutation status can influence prognosis and management in AML. Accordingly, clinical testing (i.e., RT-PCR, NGS and IHC) for mutant *NPM1* is increasing in order to detect residual disease in AML, alongside flow cytometry (FC). However, the relationship of the results from RT-PCR to traditional NGS, IHC and FC is not widely known among many practitioners. Herein, we aim to: i) describe the performance of RT-PCR compared to traditional NGS and IHC for the detection of mutant *NPM1* in clinical practice, and also compare it to FC, and ii) provide our observations regarding the advantages and disadvantages of each approach in order to inform future clinical testing algorithms.

**Methods:**

Peripheral blood and bone marrow samples collected for clinical testing at variable time points during patient management were tested by quantitative, real-time, RT-PCR and results were compared to findings from a Myeloid NGS panel, mutant NPM1 IHC and FC.

**Results:**

RT-PCR showed superior sensitivity compared to NGS, IHC and FC with the main challenge of NGS, IHC and FC being the ability to identify a low disease burden (<0.5% NCN by RT-PCR). Nevertheless, the positive predictive value of NGS, IHC and FC were each ≥ 80% indicating that positive results by those assays are typically associated with RT-PCR positivity. IHC, unlike bulk methods (RT-PCR, NGS and FC), is able provide information regarding cellular/architectural context of disease in biopsies. FC did not identify any *NPM1*-mutated residual disease not already detected by RT-PCR, NGS or IHC.

**Conclusion:**

Overall, our findings demonstrate that RT-PCR shows superior sensitivity compared to a traditional Myeloid NGS, suggesting the need for “deep-sequencing” NGS panels for NGS-based monitoring of residual disease in *NPM1*-mutant AML. IHC provides complementary cytomorphologic information to RT-PCR. Lastly, FC may not be necessary in the setting of post-therapy follow up for *NPM1*-mutated AML. Together, these findings can help inform future clinical testing algorithms.

## Introduction

Nucleophosmin (*NPM1*) is a highly expressed nucleolar protein, which has been implicated in diverse cellular functions in many cell types (1). Genetic alterations including translocations and mutations of *NPM1* have been reported in a variety of myeloid disorders (1, 2). More specifically, mutations in *NPM1* have been reported in approximately 50% of acute myeloid leukemia (AML) with a normal karyotype and represents a distinct subset of AML according to the World Health Organization classification (2, 3). *NPM1* mutations in AML are frameshift variants (i.e., small insertion/deletions) in the terminal exon, frequently involving codons Trp288 or Trp290 (3). The most common variant is the Type A mutation (c.860_863dupTCTG; p.W288Cfs*12), which constitutes approximately 80% of all *NPM1* mutations in AML (3). As a result of the Type A frameshift variant (as well as other less common frameshift variants), the amino acid sequence of the C-terminus of the NPM1 protein is altered, leading to abnormal cytoplasmic localization of mutant NPM1 in leukemic cells (3, 4).

The presence of *NPM1* mutations in AML has prognostic significance which is modified with co-mutations in *FLT3* (internal tandem duplications, ITD) and *DNMT3A*. For example, *NPM1* mutation without *FLT3* ITD or *DNMT3A* co-mutation tends to be associated with a better prognosis, than when co-existing with *FLT3* ITD and/or *DNMT3A* mutations (2, 5–8). Thus, the mutation status of *NPM1* (as well as other genes such as *FLT3*) can help direct clinical management decisions related to bone marrow transplantation (2, 9, 10) as well as specific chemotherapy regimens, including the use of BH3-mimetics (e.g., venetoclax) (11–13). Additionally, *NPM1* has been shown to be a reliable molecular biomarker of disease, which is retained in > 90% of relapsed patients who initially had mutated *NPM1* at AML diagnosis (9, 14, 15); thus, interrogation of mutant *NPM1* can be used to identify minimal/measurable residual disease (MRD) and/or early relapse, post-therapy. According to Ivey et al., after the second cycle of chemotherapy, AML patients with mutated *NPM1* transcripts detectable in the blood had a greater relapse rate and shorter survival than patients without detectable mutated *NPM1* transcripts (9). Other studies have suggested a relationship between high *NPM1* mutant allele burden at diagnosis and inferior clinical outcome in *de novo NPM1*-mutated AML (16–18). Additionally, recent preliminary studies suggest that therapy response (i.e., post-therapy fold-reduction of mutant *NPM1* transcripts from baseline levels at diagnosis) may affect risk of disease progression (19). Thus, the need to assess and track mutant *NPM1* is growing, as part of an overall effort to harness molecular and immunophenotypic approaches to evaluate residual disease and identify early relapse in AML (20, 21).

Molecular testing for *NPM1* mutation has been described using both RNA and/or genomic DNA (15, 22). RNA has been suggested to provide greater analytical sensitivity than genomic DNA, and consequently, RNA input has been used in most follow-up monitoring studies (9, 22). Typically, RNA is used in a quantitative, real-time, reverse transcription PCR (RT-PCR) assay using mutant-*NPM1* specific primers with the *NPM1*-mutant transcript signal normalized by *ABL1* transcripts (% Normalized Copy Number: (mutant *NPM1* copy number/*ABL1* copy number) x 100). Other methods to detect mutant *NPM1* including next generation sequencing (NGS)-based (23, 24) and immunohistochemical (IHC)-based (17, 25, 26) approaches have been described. A recent European LeukemiaNet consensus document has begun to raise awareness of the clinical and technical challenges for the application of molecular (i.e., RT-PCR, NGS) and flow cytometric (FC) methods to assess MRD in AML, including the assessment of mutant *NPM1* (21). Thus, as these assays become more broadly implemented in the clinical setting, continued improvement in the understanding of the relative performance of these assays is needed by practitioners in pathology, oncology and other disciplines who perform, interpret and use the results for clinical management. Currently, RT-PCR is considered by most to be the “gold standard” for the detection of MRD in *NPM1*-mutant AML; thus, at our center, we have implemented RT-PCR, alongside other testing methods, to assess mutant *NPM1* as a part of prospective testing used in real-time, during routine clinical practice. Herein, we aim to: *i*) compare the performance of our RT-PCR assay to our NGS and IHC (i.e., to compare the detection of mutant *NPM1* RNA transcript, mutant *NPM1* genomic DNA sequence, and mutant NPM1 protein, respectively) as well as compare RT-PCR to routine FC, *ii*) provide information regarding the advantages and disadvantages experienced with each approach.

## Materials and Methods

### Cases

The samples comprise individual, consecutively received peripheral blood (PB) and/or bone marrow (BM) specimens from patients undergoing routine clinical work up for diagnosis and/or follow up of acute myeloid leukemia [94 samples from 37 unique patients: 17 men, 20 women, average age at diagnosis: 60y (range 30-93y)]. Patients had a prior history of *NPM1* mutation (Type A) and corresponded to follow up samples, except for 7 samples (#21, #28, #62, #63, #66, #84, #93) which were tested at initial diagnosis (of note, all of these 7 initial diagnostic samples showed concordant results for *NPM1* mutation status by RT-PCR, NGS and IHC (pan-negative: #21, #28, #62, #63, #66, #93; or pan-positive: #84). Appropriately, the 6 samples that were negative for *NPM1*-mutation at diagnosis (#21, #28, #62, #63, #66, #93) were not used in the comparison of FC to RT-PCR since, as expected, FC would be positive for leukemia at diagnosis and RT-PCR would be negative. Overall, all samples with *NPM1* RT-PCR data available and data from at least one other testing modality (NGS, IHC or FC) were included (see **Supplementary Table 1** for data available for each sample). Specimen types available for RT-PCR and NGS included both PB and BM, while specimen type available for IHC and FC included only BM samples (**Supplementary Figure 1**). In standard clinical practice, all laboratory tests are not performed on both PB and BM simultaneously. Thus, for PB specimens with RT-PCR data or with RT-PCR and NGS data available, comparison was made to the results of the other testing modalities (e.g., IHC, FC) from a recent BM biopsy (within 2 weeks of the PB sample). In addition, separate comparison of the results from the various testing modalities was performed after restricting samples to the same specimen type and the same collection date (**Supplementary Table 2**), in order to rule out any potential confounding effect of time and sample for the assay results being compared. Lastly, a separate group of diagnostic AML samples which did not have RT-PCR data were used to compare mutant NPM1 IHC *versus* NGS (**Supplementary Table 3**).

### Real-Time, Reverse Transcription PCR (RT-PCR) Assay for Type A Mutant *NPM1* Transcripts

Patients’ PB and/or BM aspirate samples collected in EDTA tubes underwent RNA extraction (QIAamp RNA Blood Mini Kit, #52304) according to standard protocol. Reverse transcription was performed with RNA (≥ 1µg) in 20 µL final volume (SuperScript III RT, Life Technologies, #18080085; RNaseOUT, Life Technologies, #10777019; Random Primers, Life Technologies, #48190011; dNTP mix (10 mM), Promega, #U151B; First Strand Buffer (5x) and DTT (0.1 M)) at 65°C for 5 minutes, then placed on ice or left at 4°C for at least 1 minute; cycling conditions: 25°C (10 min), 50°C (50 min), 85°C (5 min), 4°C (Hold) (Applied Biosystems, GeneAmp 9700 thermocycler or ProFlex PCR systems). cDNA was diluted by adding 30 µL of nuclease-free water (50 µL total volume). Five µL of the diluted cDNA (corresponding to ≥ 100 ng RNA equivalent) was input into real-time PCR (*NPM1* mutA MutaQuant primers and probes, Ipsogen, Qiagen, #677513) using ABI 7500 Fast Real-Time PCR system (Applied Biosystems), TaqMan Universal PCR Master Mix, Life Technologies, #4304437; with cycling conditions: 50°C (2 min); 95°C (10 min); [95°C (15 sec), 60°C (60 sec)] x 50 cycles. Analysis was performed with a threshold of approximately 0.1 set for both *NPM1* and *ABL*. RT-PCR results were reported as % Normalized Copy Number (%NCN): (*NPM1* Mutant A Copy Number/ABL Copy Number) x 100. All samples and controls were run in duplicate reactions. We have previously validated the performance characteristics of the RT-PCR assay to detect *NPM1* Type A mutant transcripts in patient PB and BM aspirate specimens (27); the RT-PCR assay showed the expected analytical sensitivity (limit of detection approximately 0.01% NCN).

### Myeloid NGS Panel

A custom 45 gene NGS panel [Thunderstorm system, RainDance Technologies, Billerica, MA, USA, Illumina MiSeq (v3 chemistry)] interrogating single nucleotide variants (SNV) and insertion/deletions (InDels) was used. Samples were run in duplicate. The NGS detection sensitivity was approximately 2% for SNV and 1% for INDEL. In addition, every NGS case was manually reviewed in IGV for the presence of any mutant *NPM1* sequencing reads (limit of detection approximately 0.1%-1% variant allele frequency (VAF)). The mean and median sequencing read depth at the *NPM1* mutation locus (chr5:170,837,530-170,837,570) were 3036 and 2905 reads, respectively.

### Immunohistochemistry

IHC for mutant NPM1 was performed on 5um Bouin or formalin fixed paraffin embedded BM core biopsy or clot sections using anti-mutant NPM1 antibody (Thermo Fisher/PA1-46356, Polyclonal, 1:1500 dilution) with ER2 antigen retrieval for 30 mins [H2 (30 mins) on the Leica Bond III automated immunostainer using alkaline phosphatase Refine Red detection (Leica Biosystems Inc., Buffalo Grove, IL.]. The antibody is directed against the epitope of Type A mutation (c.863_864insTCTG) of *NPM1* and it targets the c-terminus region (including amino acids within the final exon of *NPM1*). Differences in immunostaining were not observed for Bouin or formalin fixed samples. Immunostaining results were reviewed by the original hematopathologist and also reviewed by a separate hematopathologist (MK) blinded to results from other testing modalities. Immunostaining for mutant NPM1 protein was considered positive if there was homogenous, cytoplasmic staining in >3 hematopoietic cells, with the positive cells often forming clusters. Cases were considered negative if there was no such staining in hematopoietic cells. Cases were considered borderline positive when there were very rare (i.e., 1-3 cells), scattered hematopoietic cells with variable cytoplasmic staining, such that the result could not be easily distinguished from background signal, due to the very low number and variable intensity staining of suspected cells. If there was discordance such that one pathologist considered the case to be borderline positive by IHC, but the other pathologist considered it negative, then the case was assigned to the borderline positive IHC category, since the presence of any potential mutant NPM1 protein staining (above background) in hematopoietic cells could be significant (given that the mutant protein is not expressed in normal tissues).

### Flow Cytometry

Flow cytometry (8-color) was performed using the EuroFlow AML panels (28), which include many markers (e.g. CD45, HLA-DR, CD117, CD34, CD13, CD33, CD56, CD7, etc). FC does not include analysis of NPM1 protein. FC results were analyzed with BD FACSDiva software (BD Biosciences, San Jose, CA). Gating was performed using FSC-A/FSC-H to identify singlets, CD45/Viability Dye to identify viable cells, and FSC/SSC, CD45/SSC to identify blast, lymphocyte, monocyte and granulocyte subpopulations. In follow up samples, identification of leukemic blasts was performed by assessment for the abnormal immunophenotypic profile which was recorded at diagnosis. Approximately 500,000 cells were collected in samples if FC appeared negative and an adequate number of cells were available. FC was reported as positive when there was diagnostic evidence of the patient’s prior leukemic blast population. FC was reported as borderline when there was an atypical blast population with a dissimilar phenotype of uncertain significance. FC was considered negative when diagnostic evidence of acute leukemia was not seen.

### Statistical Analysis

Sensitivity, specificity, positive predictive value (PPV), negative predictive value (NPV) and concordance (i.e., the number of cases which were positive (and negative) by the 2 respective testing modalities, divided by total number of cases tested) were calculated for each modality (NGS, IHC and FC) in comparison to RT-PCR. Fisher’s Exact Test was used for statistical comparison (**Table 2**, **Supplementary Table 2**). PPV and NPV indicate the performance of NGS, IHC and FC compared to RT-PCR (which is considered the “gold standard” test). Statistical analysis was performed using the base R package; graphics were generated with GGPLOT2.

This work is covered under the IRB Protocol #: 1007011151.

## Results

Samples were acquired from patients undergoing routine clinical workup for diagnosis and/or follow up of AML. A total of 94 PB or BM specimens were received for *NPM1* quantitative RT-PCR testing, for which data were also available from other testing modalities (e.g., NGS, IHC, FC) (**Supplementary Figure 1**). A small subset of patients in the current cohort had both PB and BM available from the same date of collection; RT-PCR of these samples showed %NCN scores for BM which were similar to, or greater than, the % NCN score seen in the concomitant PB (**Supplementary Figure 2**), consistent with the pattern of potentially greater sensitivity of bone marrow samples reported previously (9).

*NPM1* Type A mutant RT-PCR results were compared to NGS, IHC and FC results (**Table 1**). Overall, there were 94 samples with RT-PCR data including 54 (57%) positive and 40 (43%) negative samples. Among these 94 samples, NGS data was available for 72 (77%), IHC data for 81 (86%) and FC data for 81 (86%) samples (see **Supplementary Table 1** for additional details regarding data available from each modality for each sample). Among the RT-PCR positive samples, 12 of 39 (31%) samples with NGS data were NGS positive, 37 of 47 (79%) samples with IHC data were IHC positive (including borderline positive samples), and 17 of 46 (37%) FC samples were FC positive (including borderline positive samples) (**Table 1**).

**Table 1:** Summary of Results: NPM1 Type A Mutation Status.

	RT-PCR+	RT-PCR-	Total
	(*n* = 54)	(*n* = 40)	(*N* = 94)
NGS, *n* (%)			
NGS+	12 (31%)	2 (6%)	14 (19%)
NGS-	27 (69%)	31 (94%)	58 (81%)
Total	39 (100%)	33 (100%)	72 (100%)
*NPM1* IHC, *n* (%)			
IHC+	21 (45%)	0 (0%)	21 (26%)
IHC Borderline+	16 (34%)	7 (21%)	23 (28%)
IHC-	10 (21%)	27 (79%)	37 (46%)
Total	47 (100%)	34 (100%)	81 (100%)
FC, *n* (%)			
Flow+	6 (13%)	5 (14%)	11 (14%)
Flow Borderline+	11 (24%)	4 (12%)	15 (18%)
Flow-	29 (63%)	26 (74%)	55 (68%)
Total	46 (100%)	35 (100%)	81 (100%)

In order to better understand the relationship between RT-PCR results compared to Myeloid NGS, Mutant NPM1 IHC and FC, contingency tables were generated (**Table 2**).

Table 2*NPM1* status: RT-PCR v. NGS.

**Table:** 

	PCR+	PCR-	Total	Predictive Value
NGS+	12	2	14	Positive: 86%
NGS-	27	31	58	Negative: 53%
Total	39	33	72	
	Sensitivity:	Specificity:		Concordance:
	31%	94%		60%

P Value: 0.01473.

**Table:** *NPM1* status: RT-PCR v. IHC.

	PCR+	PCR-	Total	Predictive Value
IHC+	37	7	44	Positive: 84%
IHC-	10	27	37	Negative: 73%
Total	47	34	81	
	Sensitivity:	Specificity:		Concordance:
	79%	79%		79%

P Value: 0.0000003.

**Table:** RT-PCR v. Flow Cytometry.

	PCR+	PCR-	Total	Predictive Value
Flow+	17	3	20	Positive: 85%
Flow-	29	26	55	Negative: 47%
Total	46	29	75	
	Sensitivity:	Specificity:		Concordance:
	37%	90%		57%

P Value: 0.01512.

### Myeloid NGS

**Figure 1:**
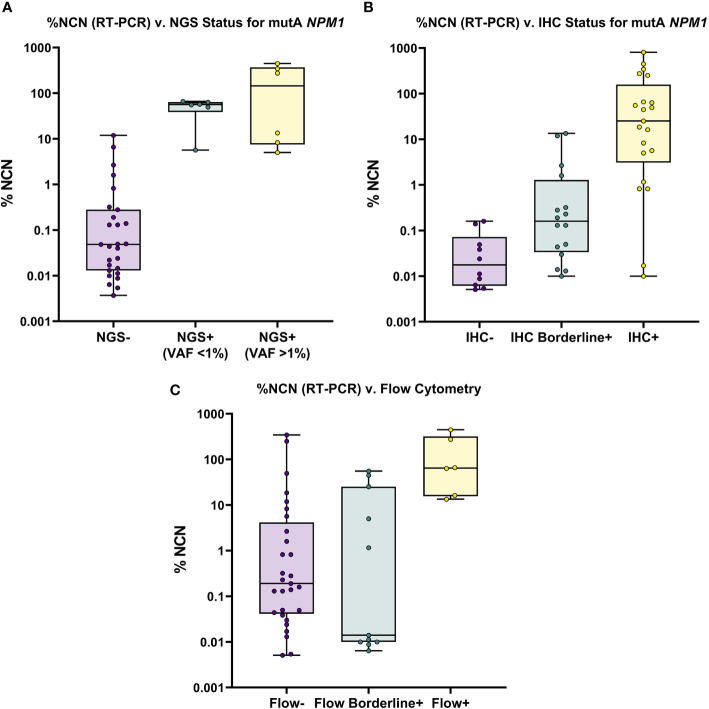
Overview of NGS, IHC and FC Results for All RT-PCR Positive Samples. **(A)** Mutant (Type A) *NPM1* RT-PCR (%NCN) (*plotted on Log_10_ scale*) is shown compared to NGS results; samples are grouped by NGS status (i.e., Negative, <1% VAF and >1% VAF). Samples are noted to be detectable by NGS only when RT-PCR % NCN values are ≥5%. **(B)** Mutant (Type A) *NPM1* RT-PCR (%NCN) is shown compared to IHC results; samples are grouped by IHC status (i.e., Negative, Borderline Positive and Positive). In general, mutant NPM1 protein is detectable (i.e., Positive) by IHC when RT-PCR % NCN values are ≥1% (although occasional samples may show detectable mutant NPM1 protein by IHC at lower % NCN values (i.e., 0.01-1% NCN). **(C)** Mutant (Type A) *NPM1* RT-PCR (%NCN) is shown compared to FC results; samples are grouped by FC status (i.e., Negative, Borderline Positive and Positive). In general, samples are Positive by FC only when RT-PCR % NCN values are >10% (although occasional samples may show borderline FC positivity at lower % NCN values).

As noted above, the Myeloid NGS panel showed a sensitivity of 31% (12/39) compared to RT-PCR; the RT-PCR positive samples which were negative by NGS had RT-PCR values of ≤ 0.5% NCN in 22 of 27 samples (**Supplementary Table 1**, **Figure 1**) indicating a low disease burden that was detectable by RT-PCR but not NGS. The remaining 5 samples had >0.5% NCN by RT-PCR and 4 of these samples had IHC data available; each of the 4 samples were positive by IHC, compatible with the positive RT-PCR result. These 27 NGS-negative cases accounted for the negative predictive value of approximately 53% for NGS compared to the RT-PCR assay. On the other hand, the Myeloid NGS panel showed a specificity of 94% (**Table 2**); the 2 samples which were positive for NGS, but negative by RT-PCR (samples 27 and 79, **Supplementary Table 1**, both with known history of NPM1 Type A mutation) showed very low/borderline NGS VAF values (approximately 0.08% VAF), borderline IHC results and negative FC results. These 2 cases resulted in the NGS assay showing a positive predictive value 86% compared to RT-PCR. For sample 27, RT-PCR and NGS were both performed on the BM specimen at 100 days post-transplant; additional follow up over the subsequent 10 months for this patient has shown persistent negativity for *NPM1* by both RT-PCR (x2) and NGS (x2) (samples 36 and 75). The underlying reason for the difference in the RT-PCR and NGS results for sample 27 is uncertain but, may have resulted from sampling variability at a low level of disease, that subsequently was associated with more complete clearance of disease further out from transplant. For sample 79, NGS was performed on BM (at 28 days post-transplant) and subsequently (i.e., 6 days later) RT-PCR was performed on PB (as a follow up for the NGS finding); subsequent follow up on this patient 2 months later has revealed BM positivity for a very low level of disease by RT-PCR (0.014% NCN, sample 92) and borderline staining by IHC.

Taken together, these findings indicate that the genomic DNA-based, standard Myeloid NGS panel used herein (with limit of detection 0.1%-1% VAF) is less sensitive than RNA-based RT-PCR, due primarily to the challenge of detecting mutant *NPM1* in samples with a low burden of disease (<0.5% NCN by RT-PCR). Nevertheless, NGS may provide some helpful information in BM cases with borderline IHC and negative RT-PCR of PB. These findings support the need for further development and implementation of “deep-sequencing” NGS-based approaches in order to improve the sensitivity of NGS-based assays for the assessment of *NPM1*.

### Mutant NPM1 IHC

**Figure 2:**
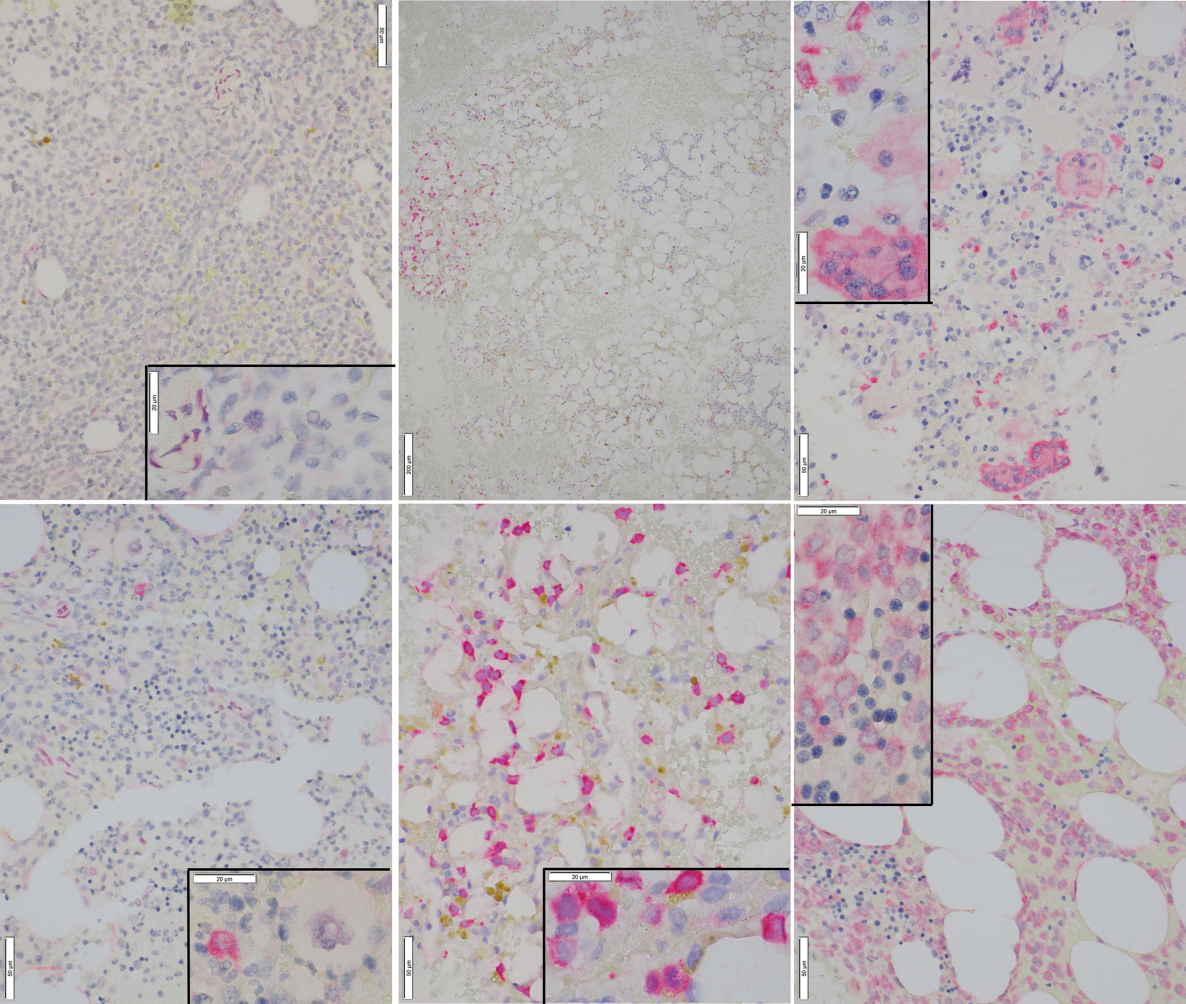
Representative Images of Mutant NPM1 IHC. *Top Left:* Bone marrow negative for mutant NPM1 immunostaining (40x); inset (100x) shows lack of staining in hematopoietic cells; occasional background staining in vascular cells is noted. *Bottom Left:* Bone marrow borderline positive for mutant NPM1 immunostaining (40x); inset (100x) shows an immature hematopoietic cell which is positive for red, homogeneous cytoplasmic, mutant NPM1 immunostaining. Adjacent megakaryocyte is negative. *Top Center:* Bone marrow positive for mutant NPM1 immunostaining; this low power view (10x) shows patchy nature of staining; the cluster of red (positive) cells is noted on the left side of field. *Bottom Center:* Bone marrow positive for red, homogeneous cytoplasmic mutant NPM1 immunostaining in hematopoietic cells(40x); inset(100x). *Top Right*: Bone marrow positive for mutant NPM1 immunostaining; scattered positive hematopoietic cells are seen admixed with scattered positive megakaryocytes (40x); inset (100x). *Bottom Right:* Bone marrow positive for mutant NPM1 immunostaining in a sample with a very high tumor cell burden; frequent positive hematopoietic cells are seen (40x); inset (100x): focally admixed mature erythroid elements (i.e., cells with small, round, hyperchromatic (i.e., dark blue) nuclei) are negative.

Representative images of immunostaining are shown in **Figure 2**. Comparison of IHC results to RT-PCR, reveals a sensitivity of 79% (37/47) was observed for the IHC (including borderline IHC positive samples)(**Table 2**); the RT-PCR positive cases which were negative by IHC (10 samples) had a very low disease burden as evidenced by the %NCN RT-PCR values (≤0.05% NCN in 8 of 10 samples and <0.5% in the remaining 2 samples (**Supplementary Table 1**, **Figure 1**); in 7 of these 10 samples NGS data was available (**Supplementary Table 1**) and no mutant *NPM1* alleles were detected in the 7 samples, consistent with the above NGS findings for cases with a low burden of disease by RT-PCR. Given the findings in these 10 samples, the negative predictive value of the IHC was 73% compared to the RT-PCR assay. In terms of other performance variables, the specificity of the IHC was 79% compared to RT-PCR (**Table 2**) and the positive predictive value of IHC was 84% compared to RT-PCR. The 7 samples (sample #s: 16, 17, 27, 37, 65, 67, 79)(**Supplementary Table 1**) which were positive by IHC but negative by RT-PCR showed only borderline IHC results, were negative by FC results, and were negative by NGS except for two cases (samples 27 and 79 which showed very low/borderline 0.08% VAF for *NPM1* Type A mutation by NGS, as described in preceding section). Overall, the underlying reasons for the difference in the RT-PCR and IHC results for these 7 samples likely includes sampling variability at a low level of disease and/or difficulty discerning background IHC staining from true, rare positive cells. Sample 79 highlights a situation where borderline IHC results (when associated with even minimal evidence of mutant NPM1 by NGS) may be relevant, since RT-PCR on a subsequent BM sample from this patient (sample 92) has revealed very low persistent levels of mutant *NPM1* by RT-PCR(0.014% NCN) as well as borderline IHC staining. Furthermore, the findings from another patient (corresponding to samples 16, 17, 37) also illustrate the potential utility of IHC in some BM samples to prompt appropriate follow up studies; more specifically, sample 16 (PB) and sample 17 (BM) collected at the same time were both negative for mutant *NPM1* by RT-PCR and NGS; FC was also negative in the BM, however, IHC showed borderline staining. A subsequent PB specimen (sample 24) was negative by RT-PCR and NGS. Continued follow up of the patient over time revealed a very low level of mutant *NPM1* transcripts (0.05% NCN) in PB (sample 33) by RT-PCR. During further follow up, BM biopsy was done and showed mutant NPM1 staining in rare, scattered cells (**Figure 2**, bottom left panel); NGS on the BM was negative and concomitant RT-PCR on a limited PB specimen (sample 37) appeared negative. Further follow up PB samples (sample 41, 42) confirmed low level of mutant NPM1 transcripts by RT-PCR. Taken together, the scenarios from these two patients highlight how mutant NPM1 IHC can be helpful in some cases to prompt appropriate follow up monitoring by RT-PCR and other studies.

A related useful aspect of mutant NPM1 immunostaining is that it can provide helpful information when the cellularity of BM aspirate and PB specimens used for bulk studies (i.e., RT-PCR, NGS and FC) may be variable (e.g., due to a “packed-marrow”) and may underestimate tumor cell number (Sample 64, **Figure 2**, bottom right panel). Again, in this situation also, the presence of mutant NPM1 staining can prompt appropriate follow up studies (RT-PCR, NGS, etc).

Lastly, IHC can provide information regarding the cytomorphologic details of mutant NPM1-positive cell types; it was observed that in addition to hematopoietic precursor-like cells (i.e., blast forms) being positive by mutant NPM1 IHC, occasional megakaryocytes were also positive. For example, a case with a subset of the megakaryocytes positive for mutant NPM1 is shown (**Figure 2**, top right panel). Subsequent biopsies from this patient (data not shown) revealed persistent mutant NPM1 staining predominantly in immature hematopoietic cells, as well as scattered megakaryocytes.

Taken together, these findings indicate that, compared to RT-PCR, mutant NPM1 IHC demonstrates sensitivity and specificity approaching 80%, and provides cytomorphologic insight into mutant NPM1-positive cell populations in biopsies, but that interpretation of IHC in samples with a low burden of disease can be challenging and thus requires correlation with molecular studies (e.g., RT-PCR).

### Flow Cytometry

In comparison to RT-PCR, FC showed a sensitivity of 37% (17/46) (including borderline FC positive samples) (**Table 2**). The RT-PCR positive cases which were negative by FC had a variable disease burden as evidenced by the %NCN RT-PCR values of ≤ 0.5% NCN in 18 of 29 cases, 0.5%-5% NCN in 4 samples, 5%-15% NCN in 3 samples and >15% NCN in 4 samples (**Supplementary Table 1**, **Figure 1**). In 7 of the 11 samples with disease burden >0.5% NCN by RT-PCR which were negative by FC, the samples available for FC were limited due to hypocellularity (mean +/- SD = 260,000 +/- 58,000 cells). Given the negative FC findings in the RT-PCR positive samples, the negative predictive value of the FC was 47% compared to the RT-PCR assay. In terms of other performance variables, the specificity of the FC was 90% (26/29) compared to RT-PCR (**Table 2**) when considering cases which had a known history of mutant *NPM1*. As shown in **Table 2** and **Supplementary Table 1**, only 3 samples with a history of mutant *NPM1* were positive by FC and negative by RT-PCR (samples # 36, 43 and 55); these samples were also negative by NGS and IHC, and, not surprisingly showed only borderline FC results (0.3%-0.7% abnormal cells by FC). These 3 cases resulted in the overall FC positive predictive value being 85%. The atypical flow cytometric findings in these 3 cases likely represented immunophenotypic variability resulting from therapy and/or presence of clonal (non-leukemic) populations; indeed, as mentioned above, NGS in each of these 3 cases was negative for *NPM1* mutation but, interestingly, 2 of these 3 samples did show variants associated with clonal hematopoiesis (i.e., *DNMT3A* and/or *TET2*). FC did not detect residual disease in any cases which was not identified by RT-PCR, NGS or IHC. Taken together, the FC findings indicated that FC is less sensitive than RT-PCR, and phenotypic variations post-therapy related to clonal hematopoiesis may confound assessment for residual disease by FC. Thus, in the setting of monitoring for residual disease in *NPM1*-mutant AML, FC appears to provide no additional benefit beyond RT-PCR, NGS and IHC.

In addition to the above findings regarding RT-PCR, NGS, IHC and FC results, a similar pattern of findings was observed when comparing the available RT-PCR, NGS, IHC and FC results for samples restricted to the same specimen type and the same collection date (i.e., PB samples with RT-PCR and NGS from same date, or BM samples with RT-PCR, NGS, IHC and/or FC from same date) (See (**Supplementary Tables 1** and **2**).

### Clinical Application

**Figure 3:**
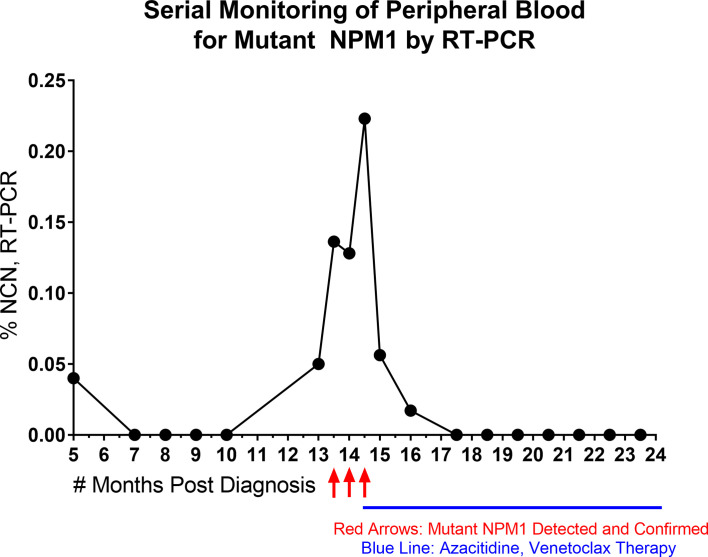
Time Course of Serial, Quantitative RT-PCR of Peripheral Blood. *NPM1* (Type A) mutant transcript monitoring by RT-PCR (%NCN) from peripheral blood is shown over time for a patient. Red arrows indicate detection and confirmation of unexpected increase in *NPM1* mutant transcripts. Blue line indicates initiation and duration of azacitidine/venetoclax therapy. Shortly after the initiation of azacitidine therapy, the *NPM1* mutant transcripts continued to rise, but then decreased during further into azacitidine treatment and remained undetectable with continuation of azacitidine/venetoclax therapy.

Lastly, to provide a specific example of the sensitivity of RT-PCR and its suitability for quantitative, serial monitoring on PB specimens, a time course of RT-PCR data from a patient is shown (**Figure 3**). The patient presented as a 58-year-old woman with an incidental finding of circulating blasts in PB. She had a history of prior gynecological tumor treated with surgical resection (no chemotherapy and no radiation therapy). Work-up lead to a diagnosis of AML with normal karyotype; molecular studies (Myeloid NGS) revealed variants in *NPM1* (Type A mutation, p.Trp299Cysfs*12, 40% VAF), *NRAS* (p.Gly13Asp, 42% VAF) and *IDH2* (p.Arg140Gln, 45% VAF). The patient was treated with induction and consolidation chemotherapy (4 cycles) and achieved complete remission. RT-PCR testing of PB for mutant *NPM1* was negative through 10 months post diagnosis, except for one borderline finding at 5 months post diagnosis (before starting final cycle of consolidation therapy), which was followed by several negative RT-PCR PB samples (as well as a BM biopsy (sample 17) at 7 months post diagnosis which was negative by RT-PCR, NGS and FC). 13 months post diagnosis, a PB sample (Sample 33) showed a low level of mutant *NPM1* transcripts by RT-PCR (0.05% NCN) and a BM biopsy was performed and was negative by NGS and FC assessment; mutant NPM1 staining revealed rare, single, scattered positive cells consistent with a borderline positive IHC result (**Figure 2**, bottom left panel)(i.e., very rare mutant NPM1 positive cells seen). Close interval monitoring of PB by RT-PCR was initiated and confirmed the presence and increase in mutant *NPM1* transcripts over the following 4 weeks. Azacitidine was started, and although the RT-PCR-detected mutant NPM1 transcripts in PB continued to rise 2 weeks after starting the azacitidine, the mutant transcript level began decreasing by day 29 after initiation. Azacitidine therapy was continued and venetoclax therapy was added. By RT-PCR, mutant *NPM1* transcript levels in the PB continued to decrease and have remained undetectable during continued azacytidine/venetoclax therapy. Taken together, this patient’s time course illustrates the potential significance of very low level (i.e., 0.05%-0.5% NCN) of mutant *NPM1* transcripts detected in PB by RT-PCR in some cases, and highlights the utility of serial, quantitative RT-PCR testing of the PB for mutant *NPM1* during patient management. Recent findings by other authors have also demonstrated the utility of quantitative RT-PCR and IHC in clinical management (29).

## Discussion

The detection and monitoring of mutant *NPM1* in AML is becoming increasingly implemented in the clinical setting. Thus, clinical practitioners in oncology and pathology need to be familiar with the various issues related to the application and interpretation of the assays used to detect mutant *NPM1* in clinical practice. Several methods are available to detect mutant *NPM1* including: RT-PCR for mutant *NPM1* transcripts (15, 21, 22), NGS for *NPM1* mutation (16, 23) and IHC for mutant NPM1 protein (17, 25). Having implemented RT-PCR for Type A mutant *NPM1* transcripts, IHC for mutant NPM1 protein and a myeloid NGS panel at our academic center, we set out to: *i*) assess the relative performance of these assays and, *ii*) provide information regarding the advantages and disadvantages of each approach.

We have found that RT-PCR for Type A mutant *NPM1* transcripts showed superior sensitivity compared to NGS, IHC and FC. Nevertheless, the positive predictive value of NGS, IHC and FC were each > 80% indicating that positive results by those assays are typically associated with RT-PCR positivity. The NGS and FC were limited in their ability to detect a low disease burden (i.e., <0.5% NCN by RT-PCR). For NGS, although manual review of the relevant NPM1 sequencing reads permitted the detection of 0.1%-1% VAF in this study, the myeloid NGS assay used herein does not incorporate molecular barcoding and is not a so-called “deep-sequencing” NGS panel. The superior analytical sensitivity of RT-PCR may also be derived from the fact that *NPM1* transcripts are known to be expressed at high levels (30, 31), and that skewed expression of *NPM1* transcripts from the mutant allele (32), lead to a technical advantage of approaches using RNA-based input. Other studies of mutant *NPM1* have also suggested that RNA-based input provides greater sensitivity than genomic DNA input (22). For FC, analytical sensitivity was impaired when the total number of cells available for analysis was limited due to sample hypocellularity. Also, for FC, the lack of an aberrant immunophenotype of the blasts in some cases can render their identification difficult by FC (21). IHC (79%) was more sensitive than NGS (31%) and FC (37%), however, samples which were negative by IHC showed a low burden of disease by RT-PCR (i.e., < 0.5% NCN). A technical limitation of IHC is that samples with low %NCN by RT-PCR often show rare, if any, scattered mutant NPM1 positive cells. Thus, a challenge with IHC can be differentiating background staining from rare, scattered mutant NPM1-positive cells. Indeed, similar findings have been recently reported by Falini et al. (29) who described rare NPM1 cytoplasmic positive cells (likely post-mitotic cells) even in occasional normal bone marrow samples. Nevertheless, in occasional cases with low burden of disease by RT-PCR (<0.5% NCN), it is possible to identify mutant NPM1 staining in rare single hematopoietic cells. Overall, IHC for mutant NPM1 alone is not suitable as a stand-alone assay for assessment of residual disease, but, in combination with RT-PCR, IHC can help visualize the tumor cell burden and provide information regarding neoplastic cell distribution and cell type; along these lines, mutant NPM1 staining was noted in occasional megakaryocytes, which has been previously described, indicating multilineage involvement of mutant NPM1 (25).

With regards to the advantages and disadvantages of each approach:

RT-PCR, in addition to its superior sensitivity, has the advantages that it is quantitative and can be performed on PB, without BM biopsy, providing non-invasive serial testing during follow up to monitor disease dynamics. However, a disadvantage of RT-PCR for *NPM1* is that mutation-specific assays are required, and up to 20% of *NPM1* mutations in AML can be non-Type A mutations (i.e., so called Type B, Type D mutations, etc.). Recently a multiplex digital droplet RT-PCR assay has been reported which permits the detection of several types of *NPM1* mutations (e.g., Type A, B, D, etc) in one reaction (33); although the specific type of *NPM1* variant present is not identifiable with that assay and confirmation of reliable performance in the clinical setting is needed. A disadvantage of all RT-PCR approaches is that the input RNA may be degraded if not handled properly during collection, transport and processing.

NGS has the advantages that it uses genomic DNA (i.e., a stable substrate), it is quantitative and can detect various *NPM1* mutation types as well as co-mutated genes (e.g., *DNMT3A*, *FLT3*, etc). However, myeloid NGS approaches in routine clinical practice often have limited analytical sensitivity, with limits of detection in the 1-5% VAF range. RT-PCR may show 1-2 orders of magnitude greater signal than NGS assays with genomic DNA input [see **Supplementary Table 1** and (22)]. More recent so-called “deep sequencing” NGS approaches (23, 24, 34) demonstrate limits of detection of approximately 0.001%-0.01% VAF. However, they typically interrogate a limited panel of genes, require higher DNA input, use increased replicates, and incur greater cost to acquire and to analyze the deeper sequencing (i.e., 200,000-450,000 reads); therefore, these “deep-sequencing” NGS approaches will likely take more time before they are widely implemented for clinical testing.

FC has the potential advantages of quantitation and cell-sorting, but its main disadvantage is the challenge of phenotypic variability in AML due to disease heterogeneity (21), therapy-induced changes in protein expression, and phenotypic aberrations associated with background clonal hematopoiesis, which can confound the identification of residual disease, as we, and others (35) have observed. Thus, in the setting of post-therapy follow up for NPM1-mutated disease, priority can be given to RT-PCR, NGS and IHC testing.

IHC has unique advantages including its capacity to visualize tumor cell burden *in situ*, and to detect architectural and cytologic features that are not apparent by bulk methods (i.e., RT-PCR, NGS, FC). In addition, IHC is inexpensive and rapid compared to the other techniques. IHC may be helpful in cases where fibrosis, a “packed-marrow” with high disease burden, or other technical factors compromise the BM aspirate material for RT-PCR, NGS and FC. However, the disadvantages of IHC include the need for a BM biopsy, its qualitative nature, and the potential difficulty to discern rare mutant NPM1-positive cells from background staining [a similar challenge has been recently reported by other authors (29)]. An additional caveat is that mutant NPM1 IHC may rarely detect cytoplasmic NPM1 in AML cases with a negative molecular assay; this may occur if the mutation involving NPM1 is not covered by a “type-specific” RT-PCR assay or occurs in an exon not covered by NGS (36). Taken together, the advantages and disadvantages mentioned above for each assay highlight how the various methods provide complementary information during the analysis of patient samples.

Our findings have prompted us to consider further optimizing our testing algorithms. Currently, we perform Myeloid NGS (along with ancillary *FLT3*, *IDH1/2* molecular testing) and FC on BM biopsies at the time of initial diagnosis of AML. We are not routinely performing RT-PCR for *NPM1* mutation status at initial diagnosis. However, given the evolving role to monitor mutant *NPM1* levels to check response to initial chemotherapy (19), and given the usual 2 week turn-around time of NGS reporting, IHC staining at initial AML diagnosis could aid in the rapid identification of *NPM1*-mutated AML cases needing baseline RT-PCR. In the follow up setting for *NPM1*-mutated AML, currently we receive PB samples for mutant *NPM1* RT-PCR monitoring. When a BM biopsy is collected, the BM is tested by NGS (for mutation status of *NPM1* and co-mutations in other genes), RT-PCR, mutant-NPM1 IHC and FC. Given the FC findings herein, we have considered eliminating FC assessment in follow up BM samples of known *NPM1*-mutated AML, if there is no other clear indication to perform FC. An additional point for further study in the setting of follow-up testing includes performance of RT-PCR on PB and/or BM samples; we (see **Supplementary Figure 2**) and others (9) have found BM samples appear to provide the potential for greater sensitivity for RT-PCR than PB.

In sum, our findings indicate that each method (RT-PCR, NGS, IHC and FC) provides complementary information and thus, while cognizant of the strengths and limitations of each assay, a multimodal assessment of mutant *NPM1* can improve our understanding of mutant *NPM1* status in patient samples.

## Data Availability Statement

The original contributions presented in the study are included in the article/**Supplementary Material**. Further inquiries can be directed to the corresponding author.

## Ethics Statement

The studies involving human participants were reviewed and approved by Weill Cornell Medicine, Internal Review Board. Written informed consent for participation was not required for this study in accordance with the national legislation and the institutional requirements. Written informed consent was not obtained from the individual(s) for the publication of any potentially identifiable images or data included in this article.

## Author Contributions

AL and MK contributed to the conception and design of the study, organized the database, performed the statistical analysis, created the figures and tables and wrote the first draft of the manuscript. SP, JG, AC, PS, GI, MG, AG-A, SL, ER, GR and WT reviewed the manuscript and provided input for edits of the initial draft. All authors contributed to the article and approved the submitted version.

## Funding

This work was performed with support from the Department of Pathology and Laboratory Medicine, Weill Cornell Medicine.

## Conflict of Interest

The authors declare that the research was conducted in the absence of any commercial or financial relationships that could be construed as a potential conflict of interest.

## Publisher’s Note

All claims expressed in this article are solely those of the authors and do not necessarily represent those of their affiliated organizations, or those of the publisher, the editors and the reviewers. Any product that may be evaluated in this article, or claim that may be made by its manufacturer, is not guaranteed or endorsed by the publisher.

## Supplementary Material

Supplementary Figure 1 Specimen Information. **(A)** Overview of specimen types available for each assay. **(B)** For each sample number, the details of specimen types available for each assay are shown.

Supplementary Figure 2 Mutant (Type A) *NPM1* RT-PCR (Peripheral Blood *vs.* Bone Marrow). Comparison of RT-PCR %NCN values obtained with paired peripheral blood and bone marrow samples collected on the same day. Overall, bone marrow specimens were noted to show similar or higher %NCN values compared to peripheral blood. Specific sample numbers are shown. %NCN = (*NPM1* Type A mutant transcript copy number/ABL copy number) x 100.

Supplementary Table 1 RT-PCR (%NCN), NGS (%VAF), IHC (% mutant NPM1 positive cells) and FC (% abnormal cells) are shown (when available) for each sample.

Supplementary Table 3 IHC for Mutant-NPM1 was performed in Diagnostic AML cases and the IHC results were compared to the results of NGS. Mutant-NPM1 IHC showed 100% concordance with NGS studies for NPM1 mutation status. IHC detected pathogenic mutations in NPM1 including Type A, Type B and pathogenic Non-Type A/Non-Type B/Non-Type D frameshift variants occurring at the known mutational hotspot region. RNA for RT-PCR was not available for these Diagnostic AML samples.
