# Supplementary material for: Comparison of Multiple Clinical Testing Modalities for Assessment of NPM1-Mutant AML
Source: Front Oncol. 2021 Aug 30;11:701318. doi: 10.3389/fonc.2021.701318 (PMC8435844; doi:10.3389/fonc.2021.701318)
Supplement: Supplementary Figure 1 — Specimen Information. (A) Overview of specimen types available for each assay. (B) For each sample number, the details of specimen types available for each assay are shown. [file DataSheet_1.zip › Supplementary Table 2.docx]

**Supplemental Table 2.**

***NPM1* status: RT-PCR v. NGS**

|  | PCR+ | PCR- | Total | Predictive Value |
| --- | --- | --- | --- | --- |
| NGS+ | 6 | 1 | 7 | Positive: 86% |
| NGS- | 19 | 22 | 41 | Negative: 54% |
| Total | 25 | 23 | 48 |  |
|  | Sensitivity: | Specificity: |  | Concordance: |
|  | 24% | 96% |  | 58% |

P Value: 0.09946

***NPM1* status: RT-PCR v. IHC**

|  | PCR+ | PCR- | Total | Predictive Value |
| --- | --- | --- | --- | --- |
| IHC+ | 15 | 2 | 17 | Positive: 88% |
| IHC- | 6 | 19 | 25 | Negative: 76% |
| Total | 21 | 21 | 42 |  |
|  | Sensitivity: | Specificity: |  | Concordance: |
|  | 71% | 90% |  | 81% |

P Value: 0.0000929

**RT-PCR v. Flow Cytometry**

|  | PCR+ | PCR- | Total | Predictive Value |
| --- | --- | --- | --- | --- |
| Flow+ | 8 | 1 | 9 | Positive: 89% |
| Flow- | 13 | 17 | 30 | Negative: 57% |
| Total | 21 | 18 | 39 |  |
|  | Sensitivity: | Specificity: |  | Concordance: |
|  | 38% | 94% |  | 64% |

P Value: 0.02324
