# Supplementary material for: Comparison of Multiple Clinical Testing Modalities for Assessment of NPM1-Mutant AML
Source: Front Oncol. 2021 Aug 30;11:701318. doi: 10.3389/fonc.2021.701318 (PMC8435844; doi:10.3389/fonc.2021.701318)
Supplement: Supplementary Figure 1 — Specimen Information. (A) Overview of specimen types available for each assay. (B) For each sample number, the details of specimen types available for each assay are shown. [file DataSheet_1.zip › Supplementary Table 3.docx]

| **Dx Case #** | **Mutant-NPM1 IHC** | ***NPM1* Mutation Status per NGS** |
| --- | --- | --- |
| Dx1111_1A | Positive | Positive: *NPM1*, c.863_864insTCTG, p.Trp288Cysfs*12 |
| Dx1111_1B | Negative | Negative |
| Dx1111_1C | Negative | Negative |
| Dx1111_1D | Positive | Positive: *NPM1*, c.863_864insCTTG, p.Trp288Cysfs*12 |
| Dx1111_1E | Positive | Positive: *NPM1*, c.863_864insTCTG, p.Trp288Cysfs*12 |
| Dx1111_1F | Positive | Positive: *NPM1*, c.863_864insTCTG, p.Trp288Cysfs*12 |
| Dx1111_1G | Negative | Negative |
| Dx1111_1H | Positive | Positive: *NPM1*, c.863_864insTCTG, p.Trp288Cysfs*12 |
| Dx1111_1I | Negative | Negative |
| Dx1111_1J | Negative | Negative |
| Dx1111_1K | Positive | Positive: *NPM1*, c.864_866delinsCCGGGCG, p.Trp288Cysfs*12 |
| Dx1111_1L | Positive | Positive: *NPM1*, c.863_864insTCTG, p.Trp288Cysfs*12 |
| Dx1111_1M | Negative | Negative |
| Dx1111_1N | Negative | Negative |
| Dx1111_1O | Negative | Negative |
| Dx1111_1P | Negative | Negative |
| Dx1111_1Q | Positive | Positive: *NPM1*, c.863_864insTCTG, p.Trp288Cysfs*12 |
| Dx1111_1R | Positive | Positive: *NPM1*, c.863_864insTCTG, p.Trp288Cysfs*12 |
| Dx1111_1S | Positive | Positive: *NPM1*, c.863_864insTCTG, p.Trp288Cysfs*12 |
| Dx1111_1T | Positive | Positive: *NPM1*, c.863_864insTCTG, p.Trp288Cysfs*12 |
| Dx1111_1U | Negative | Negative |
| Dx1111_1V | Positive | Positive: *NPM1*, c.863_864insTCTG, p.Trp288Cysfs*12 |
| Dx1111_1W | Positive | Positive: *NPM1*, c.863_864insCATG, p.Trp288Cysfs*12 |
| Dx1111_1X | Negative | Negative |
| Dx1111_1Y | Negative | Negative |
| Dx1111_1Z | Positive | Positive: *NPM1*, c.863_864insTCTG, p.Trp288Cysfs*12 |
| Dx2222_2AA | Negative | Negative |
| Dx2222_2BB | Negative | Negative |

**Supplemental Table 3.**

***NPM1* Status in Diagnostic AML Cases: Mutant-NPM1 IHC v. NGS.**
