# Supplementary figures and images for: Comparison of Multiple Clinical Testing Modalities for Assessment of NPM1-Mutant AML
Source: Front Oncol. 2021 Aug 30;11:701318. doi: 10.3389/fonc.2021.701318 (PMC8435844; doi:10.3389/fonc.2021.701318)

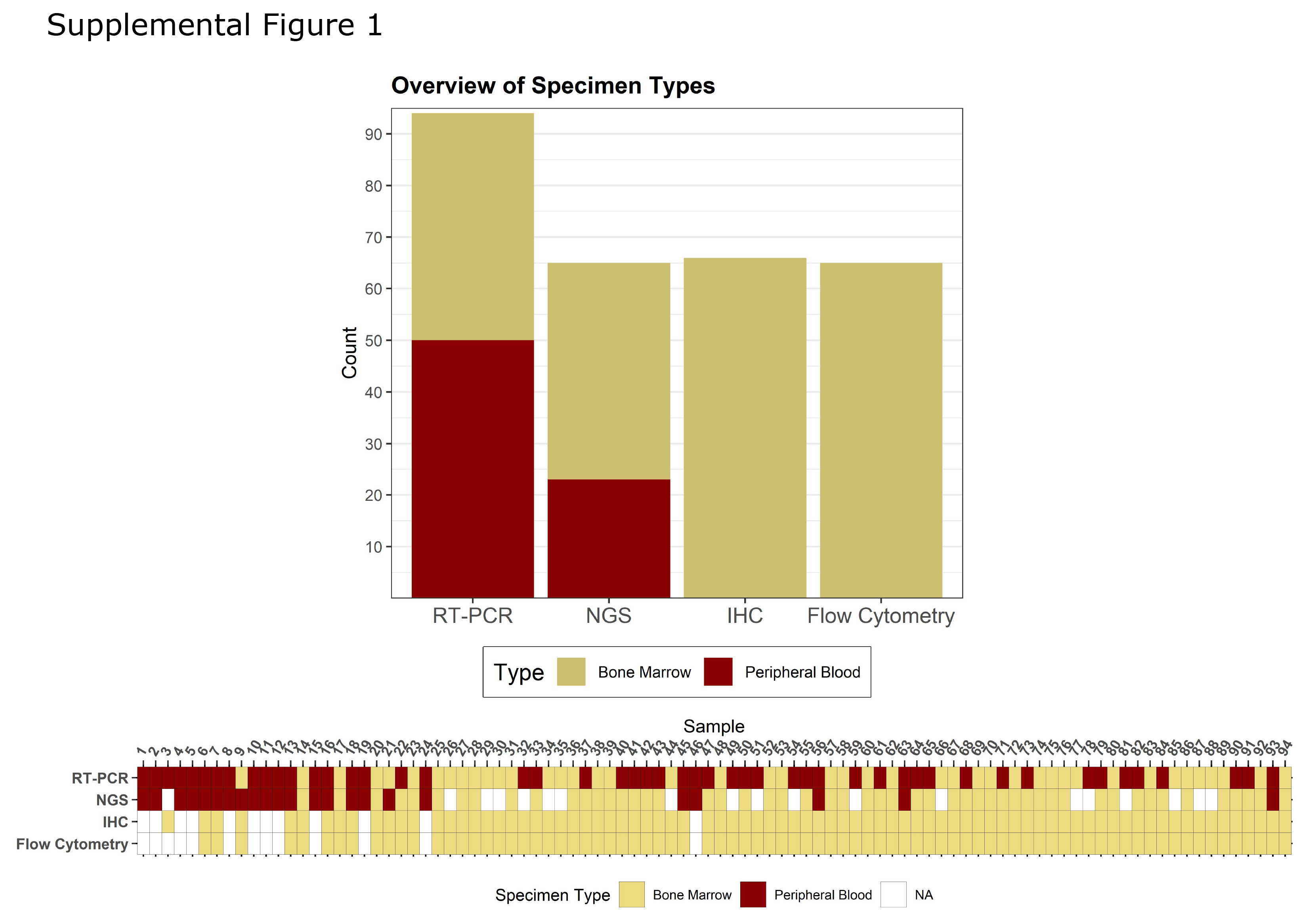

Supplement: Supplementary Figure 1 — Specimen Information. (A) Overview of specimen types available for each assay. (B) For each sample number, the details of specimen types available for each assay are shown. [file DataSheet_1.zip › Supplementary Figure 1.TIF]

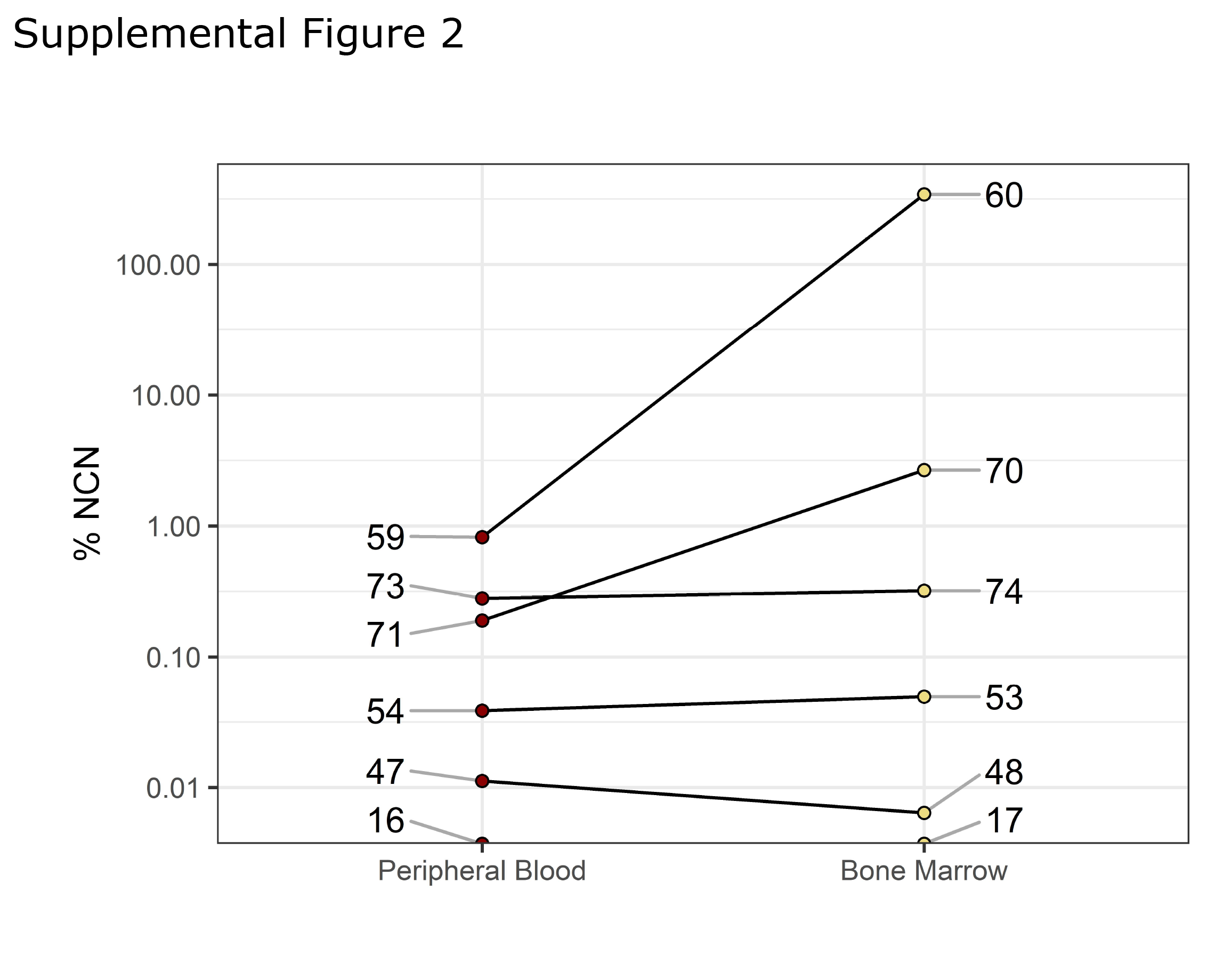

Supplement: Supplementary Figure 1 — Specimen Information. (A) Overview of specimen types available for each assay. (B) For each sample number, the details of specimen types available for each assay are shown. [file DataSheet_1.zip › Supplementary Figure 2.TIF]

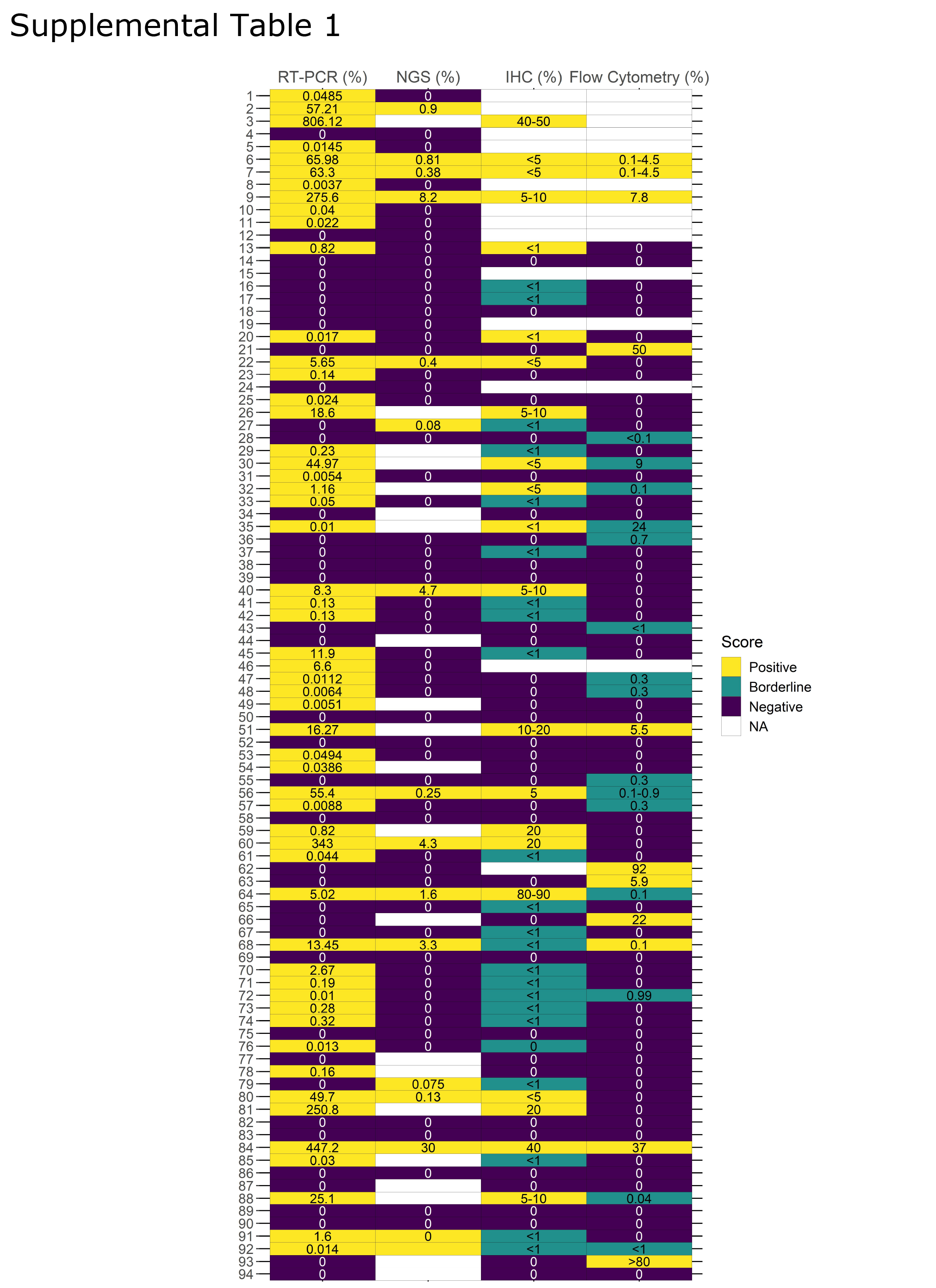

Supplement: Supplementary Figure 1 — Specimen Information. (A) Overview of specimen types available for each assay. (B) For each sample number, the details of specimen types available for each assay are shown. [file DataSheet_1.zip › Supplementary Table 1.tif]
